# Lynch syndrome: barriers to and facilitators of screening and disease management

**DOI:** 10.1186/1897-4287-9-8

**Published:** 2011-09-07

**Authors:** Kathy E Watkins, Christine Y Way, Jacqueline J Fiander, Robert J Meadus, Mary Jane Esplen, Jane S Green, Valerie C Ludlow, Holly A Etchegary, Patrick S Parfrey

**Affiliations:** 1 Centre for Nursing Studies, Eastern Regional Integrated Health Authority, St. John's, NL, Canada; 2 Clinical Epidemiology Unit, Faculty of Medicine, Memorial University of Newfoundland, St. John's, NL, Canada; 3 School of Nursing, Memorial University of Newfoundland, St. John's, NL, Canada; 4 Department of Psychiatry, Faculty of Medicine, University of Toronto, Toronto, ON, Canada; 5 Department of Genetics, Faculty of Medicine, Memorial University of Newfoundland, St. John's, NL, Canada

## Abstract

**Background:**

Lynch syndrome is a hereditary cancer with confirmed carriers at high risk for colorectal (CRC) and extracolonic cancers. The purpose of the current study was to develop a greater understanding of the factors influencing decisions about disease management post-genetic testing.

**Methods:**

The study used a grounded theory approach to data collection and analysis as part of a multiphase project examining the psychosocial and behavioral impact of predictive DNA testing for Lynch syndrome. Individual and small group interviews were conducted with individuals from 10 families with the MSH2 intron 5 splice site mutation or exon 8 deletion. The data from confirmed carriers (n = 23) were subjected to re-analysis to identify key barriers to and/or facilitators of screening and disease management.

**Results:**

Thematic analysis identified personal, health care provider and health care system factors as dominant barriers to and/or facilitators of managing Lynch syndrome. Person-centered factors reflect risk perceptions and decision-making, and enduring screening/disease management. The perceived knowledge and clinical management skills of health care providers also influenced participation in recommended protocols. The health care system barriers/facilitators are defined in terms of continuity of care and coordination of services among providers.

**Conclusions:**

Individuals with Lynch syndrome often encounter multiple barriers to and facilitators of disease management that go beyond the individual to the provider and health care system levels. The current organization and implementation of health care services are inadequate. A coordinated system of local services capable of providing integrated, efficient health care and follow-up, populated by providers with knowledge of hereditary cancer, is necessary to maintain optimal health.

## Introduction

The increased use of predictive DNA testing to determine the hereditary basis of familial cancer has important implications for cognitive, affective and behavioral outcomes of high risk individuals. Investigations into the impact of genetic testing have focused more on cognitive and affective responses and less on factors facilitating optimal disease management. Our understanding of behavioral responses is a significant gap in the research literature.

The most common hereditary colon cancer is Lynch syndrome [1-4] which is an autosomal dominant disease accounting for 2-5% of all colorectal cancers (CRCs) worldwide [1,5], with geographical clusterings observed [5,6]. A puzzling and unexplained feature of the disease is the variable expressivity (differing ages of onset, cancer sites) and incomplete penetrance (not all carriers develop the disease) [6,7]. Lynch syndrome has a lifetime CRC risk of about 80% [7,8] and is also associated with extracolonic cancers of the uterus, ovary, kidney, urinary tract, stomach, biliary tract, small intestine and brain [8]. Gynecologic cancers are important for female carriers who have a lifetime risk of 40-60% for endometrial and 10-21% for ovarian cancers [4,6].

Confirmation of Lynch syndrome means that all family members should undergo predictive DNA testing and/or be strongly encouraged to regularly screen. The effectiveness of screening in reducing morbidity and mortality from CRC is well supported [9,10]. Despite this, there is suboptimal uptake of screening by high risk individuals [11-13]. Wide variability in adherence rates have been reported, with colonoscopy screening ranging from 53-100% [11,14-20], transvaginal ultrasonography from 69-86% [14,20,21] and endometrial biopsies around 54% [21].

From a clinical management perspective, it is important to know why some high risk individuals fail to follow recommended guidelines. Few research inquiries have attempted to identify facilitators of, or barriers to, behavioral change following confirmation of hereditary disease [22-28]. Merely informing individuals of their cancer risk may not motivate behavior change [25] and could possibly impede screening if perceived to be uncontrollable [29,30].

Some authors have conjectured that awareness of familial cancer patterns and personal/family cancer experiences influence risk perceptions which, in turn, impact acceptance of a carrier status and engagement with screening [25-27,30-32]. Other authors have used social cognition theory as a template for conceptualizing cognitive and emotional factors that impact reactions to predictive DNA testing and, ultimately, behavioral responses [25,30,31]. Nevertheless, it remains unclear how risk perceptions are shaped by disease-related experiences and impact behavior.

High risk individuals are expected to manage their cancer risk [16,21,33]. This can be difficult without consensus on the scope, frequency, and age of initiation of screening for CRC [4,34-37] and extracolonic cancers [4,34,35,38]. Despite the documented benefits of prophylactic interventions, like gynecologic surgeries, for reducing cancer risk [4,34,38], these strategies have not been fully integrated into the clinical management of Lynch syndrome families.

Health care providers play a key role in encouraging high risk individuals to become involved in disease management [4,34,35,39]. It is critical that all providers are adequately informed about Lynch syndrome, obtain comprehensive medical and family histories [39-41], make referrals to genetics services [4] and recommend appropriate screening and management [3,34,36]. However, significant gaps exist in providers knowledge [12,42] and many fail to identify at-risk individuals and/or advise them appropriately [39,42].

The evidence suggests that the health care system can pose barriers to screening. Ineffectual coordination and continuity of care [43], inadequate access to and availability of screening/specialty services [44], and variation in provider recommendations [39,43] can impede effective clinical management. Currently, there is a paucity of research on how individuals interact with the health care system as they adjust to living with a confirmed hereditary cancer risk.

This article reports on findings derived from a grounded theory study on the psychosocial and behavioral impact of genetic testing on individuals at high risk for Lynch syndrome. In this paper we focus on how confirmed carriers experience disease management and view the quality of interactions with health care providers and the overall health care system. We include recommendations on how to improve disease management and facilitate quality outcomes.

## Methods

### Study design

A grounded theory study was part of a multiphase project examining the psychosocial and behavioral impact of DNA testing for Lynch syndrome. The Human Investigation Committee, Memorial University, approved the study protocol.

Grounded theory was used during data collection and analysis [45]. This approach is considered appropriate as the focus is not solely on how health threats, diagnostic procedures or treatment protocols are experienced, but also on how this information is received and assimilated into belief structures, and how this integration becomes a stimulant for actions needed to achieve optimal health functioning. The strength of this inductive approach is the emphasis placed on identifying and describing the social-psychological processes grounded in the data emerging from participant interviews [46].

### Population and predictive genetic testing

The target population was individuals from high and intermediate risk families registered in the Provincial Medical Genetics Program of Newfoundland and Labrador (NL) and participating in the larger case control study. Eligible participants for the grounded theory study were those living in families with a confirmed MSH2 mutation-the intron 5 splice site mutation (942+3A > T) (12 families) or exon 8 deletion (5 families). Details on this population have been reported elsewhere [6].

A purposive sample of 39 individuals from 10 families who had completed genetic testing and knew their status was selected from the accessible population (N = 276). Predictive DNA testing is offered to individuals in high and intermediate risk families. Follow-up counseling sessions are held with those interested in testing for known mutations. Testing results are normally reported in face-to-face sessions. Follow-up letters summarizing the results are forwarded to participants and their physicians. Clinical screening programs are adjusted according to test results.

This article focuses on 23 confirmed carriers (14 female, 9 male) from three families with the intron 5 splice site mutation and three families with the exon 8 deletion (Table 1). The mean time from genetic testing to the initial interview was 6.0 (± 2.8) years (range .1 to 9.6) and age at the first interview was 48.9 (± 13.6) years (range 26 to 78). Thirteen participants developed cancer at a mean age of 43 (± 5.8) years (range 33 to 54). Significantly, those who had reached the affected stage experienced a total of 27 primary cancers with CRC occurring at least once in 61.5% of the cases. Of the 14 female carriers, five developed endometrial cancer (35.7%) and four (28.6%) had prophylactic hysterectomies and/or oophorectomies.

**Table 1:** Participant characteristics (N = 23)

*ID*	*Family*	*Gender *	*Post-GT^a^*	*Age^b^*	*Affected*	*Onset Age*	*Cancer Types^c^*
23	1B	Female	6.42	57	No	---	---
24	1B	Female	6.42	78	Yes	46	CRC/EC
25	1B	Male	8.50	52	Yes	42	CRC/GA
26	1B	Female	2.42	32	No	---	---
31	1B	Male	7.50	47	Yes	45	CRCx2
8	1C	Male	3.67	28	No	---	---
22	1C	Male	6.50	26	No	---	---
30	1C	Male	7.42	57	No	---	---
9	2A	Female	8.08	41	Yes	39	CRCx2
32	2A	Female	9.58	69	Yes	35	CRCx2/EC/GA/SK
20	2B	Female	9.08	50	Yes	33	EC/BR/VA
21	2C	Male	9.08	50	No	---	---
10	3A	Female	7.00	42	No	---	---
27	3A	Female	8.17	51	Yes	43	Ovarian
28	3A	Male	8.17	43	Yes	45	CRC/DUO
29	3A	Female	7.50	33	No	---	---
11	3B	Male	8.42	76	Yes	39	CRCx2/SK/KD
4	6	Female	0.75	48	Yes	49	CRC
19	6	Female	0.08	63	Yes	54	SK
37	7	Female	3.17	50	Yes	46	KD/EC
38	7	Male	3.08	46	No	---	---
34	8	Female	2.83	43	Yes	40	EC
36	8	Female	3.75	42	No	---	---

Note. Families 1B to 3B have the intron 5 mutation and families 6 to 8 have the exon 8 deletion. The use of A, B or C after the family number denotes separate nuclear families within a particular extended family.

^a ^Years since genetic testing.

^b ^Age at first interview.

^c ^CRC = colorectal; EC = endometrial; GA = gastric; SK = skin; BR = breast; VA = vaginal;

KD = kidney; DUO = duodenal.

### Procedure

After initial contact, interested individuals were forwarded a cover letter, brief study summary and consent form, and re-contacted to schedule interviews. Following informed, written consent, two interviewers (principal investigator and research assistant) conducted 60 to 90 minute interviews with participants. Individual or small group interviews (immediate family only) took place in participants' homes or conference rooms. Open-ended questions elicited commentary on experiences with cancer in the family (first awareness of hereditary link, perceived personal risk, screening motivation) and genetic testing (decision-making, counseling experiences, reaction to status, understanding implications, impact on family). Additional questions evolved from the thematic content analysis (adjusting to carrier status, screening experiences, health care service needs). A second interview provided participants with an opportunity to comment upon and confirm their interpretive summaries. Information from the second interview also helped the research team augment gaps in the data, and the conceptual categories and properties of the emerging substantive theory.

### Data analysis

Data analysis proceeded in several phases. First, interviews were transcribed verbatim and perused independently by a three-member team. The focus was on interpreting the meaning of words and sentences through reading and re-reading the text, and assigning substantive codes to recurrent themes. Team discussions focused on achieving consensus on emerging themes. Second, mid-way through data collection, interviewing was temporarily stopped and the constant-comparative method of analysis applied to the data sets by two members working independently. The objective was to identify relationships between and among substantive codes. As potential category relationships were tested within the data, a substantive theory began to emerge.

Third, in-depth analysis of the first 18 transcripts revealed a family context (i.e., experiential base and degree of burden and sense of resilience), differences between carriers and non-carriers of Lynch syndrome (views of screening protocols and timelines to diagnosis, coping approaches to short/long term prognosis, implications for children) and differences between affected and unaffected carriers (intensity of reactions to cancer onset/recurrences). The focus shifted to purposive selection of an additional 14 carriers from family groupings with many (n = 9) having reached the affected stage. This approach to subject selection facilitated confirmation of the substantive codes and refinement of their properties.

In the later stages of analysis, length of time since discovery of the family-based gene mutation and the availability of and actual involvement in genetic testing surfaced as potential influencing factors on individual and family perceptions. The decision was made to sample additional individuals to determine the importance of time. Data collection continued (n = 7) until the research team was confident that the experiences of this group would not alter existing properties or categories. At the final step, the data and resulting theory were examined by an independent consultant to enhance credibility and accuracy. This resulted in a more parsimonious and refined set of themes and codes.

## Results

Data analysis revealed several personal, provider and health system barriers to and/or facilitators of effective disease management. Risk perceptions and acceptance of the genetic link to cancer influenced individuals' ability to adjust to their carrier status and accept recommended regimes. Despite the importance of risk perceptions and acceptance, interactions with the health care system and providers clearly affected overall adjustment.

### Person-Centered Barriers/Facilitators

The most important personal factors were emotional and psychosocial states, physical health status, prior experiences with cancer screening and/or treatment, and accepting the need for prophylactic interventions. These factors are categorized as risk perceptions and decision-making, and enduring screening/disease management.

### Risk perceptions and decision-making

Risk perceptions play a crucial role in motivating individuals to become involved in disease management. A meaningful balance must be forged between the cognitive and emotional spheres for decision-making. Full engagement seems to be highly contingent upon emotionally accepting potential threats to the self and understanding the benefits of ongoing monitoring and timely interventions.

Participants spoke about the emotional and physical challenges of living with Lynch syndrome. Despite understanding the importance of following recommended protocols, the burden of dealing with this disease can be overwhelming.

Like I can sit here and say to you, 'Oh yeah, all the knowledge in the world, it's great to know. But look at it from the human part of it, your own self going through this every single day'. Every time someone goes to a doctor, my crowd is like, 'Who is next, right?' It gets to you after a while. [I10, Fam3A]

All participants echoed the importance of screening while being ever mindful of the challenge of living with high cancer risk. Only one participant had not engaged in cancer screening following a positive genetic test result. However, not all of the participants were participating in the full scope of cancer screening and/or adhering to recommended intervals. Oscillating cognitive and emotional forces impinge on individuals' willingness to become fully involved in the process.

Although some participants had misgivings about knowing their status, these doubts soon subsided when screening detected cancer. Several individuals alluded to the potential benefits of regular screening.

I started seeing [gynecologist] on a regular basis. I was constantly being screened; it [uterine cancer] was picked up. I had the Pap smear and then the endometrial biopsy and both of that came back abnormal. It [cancer] was just in the early stages. [I37, Fam7]

Participants also recognized the need to accept and assume responsibility for healthy living and self monitoring for signs and symptoms of an impending illness. Some perceived this as critical for disease management.

Since I found out that I have the gene, I try to eat a little better and ... exercise a little better. You watch for things and you're a little more conscious of the things you're putting in your body. [I26, Fam1B]

How well individuals adjusted to the burden of the disease had important implications for their willingness to follow recommended guidelines. Everyone who accepted having Lynch syndrome recognized the benefits of disease management. For some, the motivation to do so was enhanced following early cancer detection.

### Enduring screening and disease management

Participants often experienced conflicting emotions about knowing what had to be done, wanting to do it and actually doing it. For many, scheduling appointments and waiting for diagnostic test results became physically draining, time consuming, and burdensome.

Successful adjustment seemed highly contingent upon living as normal a life as possible without being constantly reminded of cancer risk. The anxiety and worry associated with the probability of cancer detection created emotional barriers that impeded actions, forcing some to use "time out" periods.

I'm after falling off the wagon a bit, where I've had a couple of surgeries. ... I couldn't do one test because I was doing something else. ...Then after one of the surgeries, I guess you kind of reach your tolerance level. It was a conscious decision. ...I just had to give it up for a while. [I9, Fam2A]

Participants relayed stories of endurance and perseverance. Although the full scope of physical and emotional difficulties was individual specific and time dependent, many commented on the challenges of regular screening. Even when highly motivated, the emotional strain of upcoming procedures can be quite burdensome especially when prior experiences evoke unpleasant memories: "It's just as well to tell the truth, I cry. I'm weeks before thinking about it and I'm dreading it. I'm dreading the day that the test will come." [I37, Fam7]

For many participants, the type and frequency of screening protocols and recommended prophylactic interventions increased with evolving knowledge and/or emerging cancer patterns within the family. The increasing demands often became a struggle:

"It [screening] is cumulative and I find more and more. I don't dwell on it, but it's changing and I find I'm really, really sick of having to have this..." [I34, Fam8]

Ongoing disease management requires adequate resources to support everyday living. The significance of this for any one person can be influenced by their financial status, family responsibilities and employment history, among others. For many, accessing appropriate cancer care involves having the means and willingness to travel outside of their communities, taking time off work and/or having adequate support to deal with family responsibilities. Practical issues are important because they may interfere with one's willingness and ability to access recommended screening/treatment.

I'm a year in the hole on my sick leave here now. So if I got a flu or anything like that, I can't just stay home. Every appointment [for diagnostic tests], where I'm running to town is over so many hours ... it is sick leave. Then I had surgeries where you take off six weeks. [I9, Fam2A]

When early stage cancer is identified, physical and psychological benefits occur immediately following treatment. These benefits may not be so obvious for individuals asked to consider prophylactic surgery in the absence of signs and symptoms of disease. Female family members are encouraged to have prophylactic hysterectomy and bilateral salpingo-oophorectomy because of their high risk for endometrial and ovarian cancer, especially when parents or sisters have had these cancers. In the current study, four women had prophylactic surgery without having symptoms of disease whereas another two had hysterectomies for benign gynecological disease.

The "present" for many participants reflects a story of survivorship and endurance. It was apparent from listening to their stories that the burden of screening/treatment sometimes became a deterrent to continuance. This burden was augmented or lessened by the scope of family and work responsibilities.

### Provider-Centered Barriers/Facilitators

The perceived knowledge and skills of health care providers surfaced as key factors facilitating or impeding participation in regular screening and disease management. Participants wanted to receive care from physicians/specialists familiar with their family cancer history. Trust seemed to increase when physicians were intimately aware of the family history and acknowledged the importance of monitoring high risk cancer sites.

When you get a doctor like that [open and engaging] it means something because you don't feel like you're just a number, like they know you personally. They seem like they care and you don't come across too many like that. I felt like a number for so long. [I27, Fam3A]

Most disconcerting for participants was the perceived tendency for some physicians to discount age of onset of first cancers in families as a benchmark for screening initiation and follow-up. When physicians failed to do this, participants distrusted their knowledge: "The problem is they are young and because they are young the doctors aren't testing [screening] them properly for bowel cancer. Not testing them early enough. They're not realizing that even now after all this." [I23, Fam1B]

Integral to effective monitoring is having knowledge of the natural history of the disease. Following encounters with physicians who seemed to have limited understanding of Lynch syndrome, some participants felt the need to become better informed and share this knowledge with them.

Every time I go to him [physician] I say, 'Now do you know that these lesions are sometimes flat? ...Don't look for bumps. Look for these flat lesions which are the Lynch II'. Even now I don't know if he hears me. Because they'll always talk about removing polyps and I don't know if that's set out enough in the literature. [I20, Fam2B]

Similar concerns were expressed about physicians not perceived to be attentive enough to the extracolonic cancers.

It would be nice if we knew it was being monitored and we were all getting the proper checks. But not only just for bowel. I mean they do a colonoscopy, that's not going to show if you have anything in your ovaries or kidneys or anywhere else. [I23, Fam1B]

From a clinical management perspective, participants assessed physicians in terms of the completeness of medical care and quality of communications. Medical care was evaluated by the thoroughness of history taking and physical examinations. If unsure about a physician's approach, participants felt the need to enlighten them.

Unless you can tell a doctor what is wrong with you he can't see through you and know, unless you recognize symptoms yourself. Gone are the days when ... they [physicians] do a complete physical and chest x-ray. ...They don't look at it [cancer] as coming from a history. [I32, Fam2A]

Quality of communications was defined in terms of effective interpersonal skills. Participants wanted providers who were sincere and took the time to facilitate understanding. Some commented on the limited communication of an informative nature and the lack of perceived support: "When I go for a colonoscopy, it's the quicker you're in and out the better. It's no such thing as sit down for any discussion. We got no support system." [I25, Fam1B]. Other participants presented a contrasting perspective.

When they found things that he [specialist] has been suspicious about, he showed me the pictures and he sits down. 'This is what we are going to do'. ...So he's always been very informative. ...I appreciate that, I want that honesty. ...So I can be actively involved with what happens to me. [I21, Fam2C]

In essence, living with Lynch syndrome is an independent journey that requires being attentive to physical changes, appreciative of their implications for future health, and assertive about receiving care from knowledgeable, caring providers.

### Health Care System Barriers/Facilitators

Continuity of care at the provider and system levels seemed to pose great difficulty for participants. Continuity of care is dependent upon continuous information flow (disease and person-focused), strategic coordination of services (complementary and timely), and accessing a consistent provider mix over time. Restricted continuity of care can play havoc with successful disease management.

Especially vital is ongoing collaboration among primary and specialty care sectors during the planning and delivery of services. As the number of diagnostic procedures and potential cancer sites increase, there is a concomitant increase in the number of specialists involved in providing care and, thus, the greater potential for inconsistencies in recommended screening intervals. A couple of participants voiced their frustrations following interactions with different aspects of the health care system: "But my family doctor argued that it [colonoscopy] should be every year. I feel it should be done every year. Every three years the [specialist] wants it done." [I38, Fam7]; "I haven't been done since two years ago. That extra six months could mean a lot to me. So what am I supposed to do?" [I27, Fam3A]

Study participants were of the opinion that poor communication among providers could be detrimental to a person's well-being, quality of life and, ultimately, long-term survival. An important message conveyed is that greater consensus is needed on acceptable screening intervals and targets, especially in families with a higher than usual penetrance rate for CRC and associated cancers.

Participant comments also conveyed a vivid picture of limited organization and coordination of health care. Individuals confront challenges navigating the health care system particularly when having to deal with different institutions and physicians/specialists. At times, this requires a tenacious, persistent approach and a working knowledge of the system.

Every six months I ... have the ultrasound done. ...Then I have to make an appointment to see the specialist ... for what? It is a negative ultrasound. Then you're supposed to ... get another ultrasound but they can't get an appointment set up that far in advance. ...then you need a requisition. [I9, Fam2A]

Everyone echoed the need for a more coordinated approach that lessens the demands on personal time and coping resources. One participant commented thus, "I would like to have one stop shopping. It seems like I am running around doing all this and I don't want this. I don't need this." [I4, Fam6]

Timely access to services can become a major liability, with delays especially upsetting for individuals subject to heightened uncertainty and worry. Participants suggested that carriers should be given priority access to screening and specialty services.

After I had my operation [for colon cancer] I phoned up for another appointment [with specialist] and they told me that it could be another six months before I get in and my year was up then right. ...So I phoned the doctor that operated on me and I got in within two weeks. ...I was frightened right. [I31, Fam1B]

Despite being aware of requisite health care services, system challenges often prevented participants from 'being ahead of the game'. Especially critical is a coordinated system of care which provides timely access and follow-up. Without adequate resources, individuals are at greater risk to be burdened by the disease.

## Discussion

The current study highlights the many personal, provider and system level barriers to and facilitators of engaging in effective disease management. Study findings suggest that participants seem to be well-informed about Lynch syndrome, have accurate risk perceptions and acknowledge the benefits of regular screening. Nevertheless, the interaction of the emotional and physical burden of disease management with the practical demands of everyday living (family and work) and provider and health care system challenges may also significantly influence behavior.

Only a few studies have stressed that the behavioral impact of genetic testing is an important area for research [2,22,23,28]. Most studies have focused on psychological outcomes as opposed to potential barriers to and facilitators of informed decision-making concerning screening/treatment regimes. The current study provides informative insight into some of these factors. The findings highlight the physical and psycho-emotional obstacles (worries/concerns about potential test results/prophylactic interventions, intensity and scope of screening, preparation for and experiences with diagnostic procedures, scheduling issues) that can increase the burden of disease management. Other researchers have noted that physical and psychological barriers can add to the burden of screening, and pose deterrents to regular participation [11,14,20,32,47].

The importance of disease-related experiences for facilitating adjustment and determining the appropriateness and relevancy of healthy behaviors is not new. This finding supports, in part, the argument put forth by others that behavioral responses are a function of perceived risk which is influenced by health threat representations that continuously evolve in response to experiences with the disease in the self and/or others [25,30,31].

The current study also supports how interactions with health care providers can impact the overall burden of Lynch syndrome. Ratings of the quality of provider care are a function of perceived knowledge levels and clinical management approaches. A growing body of evidence supports the significant role played by physicians and other providers in improving adherence in this population [13,16,33,34,43]. It is therefore important that all providers become informed about current screening and treatment protocols [16,35,48].

Several authors confirm the controversy over suitable time intervals for colonoscopy [34,35,40,41] and the variable attention given to extracolonic cancers [17,38]. These inconsistencies are worrisome. Previous research has found that those at increased risk for CRC receive insufficient information on screening intervals, risk assessment and procedures, and inadequate emotional support between diagnostic tests [20,43]. This situation not only impedes development of best practice guidelines but also creates problems for physicians involved in disease management [49,50].

Experiences with and reactions to encounters with the health care system can impede effective disease management. Our findings suggest that existing counseling and disease management resources are inadequate to meet the demands that follow predictive DNA testing. An important source of dissatisfaction is gaining timely access to needed services. Ineffective coordination of diagnostic, treatment and specialists' appointments creates unnecessary delays, enhances worry, and propels some to distance themselves from the whole process. It is apparent that referral protocols need to be simplified and more coordinated. Some authors have highlighted the need for a single service [12] or a multidisciplinary team comprised of providers committed to following evidence-informed clinical guidelines [12,20,38,39,50].

Our research illuminates the possibility of new roles for health care providers in cancer genetics. In Canada there is no national registry, as provided in some smaller countries. In the province of NL four regional health authorities (RHAs) are responsible for delivering a range of health care services in hospitals, clinics and community health programs within their respective geographic areas. Recently, genetics clinics have been established in three of these RHAs. These clinics will be linked to the Provincial Medical Genetics Program which will integrate clinical care with the evaluation of interventions directed toward improving clinical outcomes. Other researchers concur that familial cancer registries and genetics service centers are perceived to be effective mechanisms for facilitating quality outcomes [12,34,35].

Despite the limitations of a small sample size and inherent biases in having participants recall how they experienced and reacted to specific events and situations, the findings do provide practical insight into barriers and facilitators that may be individual, provider and/or system based.

## Conclusions and Policy Implications

This study has further illuminated the psychosocial and behavioral impact of predictive DNA testing for Lynch syndrome. Many participants were confronted with serious issues in managing their disease. These issues require preventive strategies to help maintain optimal health and a reasonable quality of life. What is important for families is the presence of providers with the necessary knowledge and skill base and a coordinated system of local services capable of providing integrated health care and timely follow-up.

Ideally, genetic counseling should facilitate the adoption of appropriate, lifelong disease management strategies. In light of the current findings, genetic counselors may need to assess the family and socio-cultural context of hereditary cancer [24] and its potential influence on decision-making. It is also necessary to explore the emotional aspects of living with cancer risk so that the burden of the disease can be lessened.

Importantly, Lynch syndrome has significant implications for public health policy [4]. The ultimate plan should be to provide resources that enable individuals in high risk families to develop a strong sense of resilience and maintain a balanced screening schedule. In particular, this cohort requires timely and appropriate health care services, including:

○ A critical mass of genetic counselors to provide timely services to high risk families before, during and following genetic testing.

○ Service providers to coordinate and streamline diverse screening and treatment resources.

○ Health care providers, especially primary care physicians, informed about the risk of cancer within families and reinforcing the importance of maintaining recommended screening and initiating referrals to appropriate specialists.

○ Clinical monitoring tools designed to evaluate the impact of predictive testing and the ongoing psychosocial and behavioral adjustment to living in families with hereditary cancer.

The current uncoordinated, physician dependent organization of screening for individuals with Lynch syndrome in Canada is inadequate. Given the incidence and prevalence of these hereditary cancers and the clinical benefits of screening, there is a critical need to provide integrated health care and timely follow-up in a manner that facilitates navigation of and access to the health system.

## Competing interests

The authors declare that they have no competing interests.

## Authors' contributions

CW and MJE conceived and designed the study. CW and JF interviewed the participants. CW, JF, RM, VL and KW were involved in data analysis. KW and CW drafted the manuscript. PP, JG and HE critically revised the manuscript for important intellectual content. All authors read and approved the final manuscript.
